# Understanding how therapeutic exercise prescription changes outcomes important to patients with persistent non-specific low back pain: a realist review protocol

**DOI:** 10.1186/s13643-024-02466-8

**Published:** 2024-02-08

**Authors:** Lianne Wood, Vicky Booth, Sarah Dean, Nadine E. Foster, Jill A. Hayden, Andrew Booth

**Affiliations:** 1 Department of Public Health and Sports Sciences, Faculty of Health and Life Sciences University of Exeter, Exeter, UK; 2 Faculty of Medicine, University of Keele, Newcastle Under Lyme, UK; 3 Nottingham University Hospitals NHS Trust, Nottingham, UK; 4 University of Nottingham, Nottingham, UK; 5 Department of Health and Community Sciences, Faculty of Health and Life Sciences, University of Exeter, Exeter, UK; 6 Surgical, Treatment And Rehabilitation Service (STARS) Education and Research Alliance, Metro North Health and The University of Queensland, Brisbane, QLD Australia; 7 Department of Community Health and Epidemiology, Dalhousie University, Halifax, Canada; 8 School of Health and Related Research, University of Sheffield, Sheffield, UK

**Keywords:** Exercise therapy, Exercise, Lower back pain, Review

## Abstract

**Introduction:**

Persistent low back pain (LBP) is the leading cause of disability worldwide, and therapeutic exercise is recommended as a first-line treatment in international guidelines. The effects of exercise on clinical outcomes of pain and physical function are small to moderate, despite broader impacts on cardiovascular systems, biological health, mood, and emotional well-being. Therapeutic exercise prescription is defined as exercise that is prescribed by a clinician for a health-related treatment. It is unknown how therapeutic exercise prescription creates effects on outcomes of importance. Realist reviews explore how underlying mechanisms (M) may be active in the context (C) of certain situations, settings, or populations to create an intended or unintended outcome (O). Our objective is to explore and understand the mechanisms by which therapeutic exercise prescription changes outcomes for people with persistent LBP.

**Methods:**

We will develop initial programme theories based on preliminary data from a previous systematic review and consensus workshop. These theories will be modified with input from a steering group (experts), a stakeholder group (people with lived experience of exercise for persistent LBP and clinicians), and a scoping search of the published literature. An information specialist will design and undertake an iterative search strategy. These will be used to create CMO configurations, which will be refined and tested using the literature. The realist review will be reported following RAMESES guidance.

**Discussion:**

Realist reviews are uncommon in LBP research to date, yet those offer an opportunity to contrast with traditional methods of randomised controlled trials and systematic reviews and provide additional information regarding the contexts and mechanisms that may trigger certain outcomes. This can aid our understanding of the contextual features that may influence exercise prescription, such as for whom they are most effective, in what setting, how they are implemented and why. This realist synthesis will enhance our understanding of therapeutic exercise prescription to improve adherence and engagement and ultimately will provide clinically relevant recommendations regarding exercise prescription for those with persistent LBP.

**Systematic review registration:**

The review has been registered with PROSPERO (CRD42017072023).

**Supplementary Information:**

The online version contains supplementary material available at 10.1186/s13643-024-02466-8.

## Background

### Persistent low back pain

Persistent low back pain (LBP) is the leading cause of disability worldwide [1]. In 2017, it was estimated that around 577 million people were experiencing persistent LBP [1]. LBP is a symptom, not a disease, and is defined as pain between the twelfth rib and buttock creases, with or without leg pain [2]. For most people, persistent LBP is non-specific, and there is little structural pathology to explain the persistence of LBP. Many people with LBP will experience resolution within 6 weeks of onset. However, two-thirds of people with LBP report pain at 3 months and 12 months [3]. For a small proportion, LBP can persist for more than 1 year. A variety of psychological, social and biophysical factors as well as comorbidities may contribute to the presence of persistent LBP [2, 4]. Risk factors for developing persistent LBP include previous episodes of LBP [3], as well as poor mental health, work status and other chronic conditions such as asthma, headache, and diabetes [5, 6]. Persistent LBP is a multifactorial condition, influenced by both patient and practitioner beliefs, emotional status, physical activity, immune systems, genetics, work environment, co-morbidities and so forth [4].

Exercise is a frequently recommended treatment for persistent LBP [7, 8]. However, the effect of exercise on pain and disability outcomes remains small to moderate in comparison to non-exercise controls [9]. Exercise is a complex intervention with many potential treatment targets (or proposed mechanisms) [10]. Despite many high-quality randomised controlled trials (RCTs) and systematic reviews, there is still a limited understanding of how therapeutic exercise prescription changes outcomes of importance for people with persistent LBP [9]. Further, we are unclear on which components are necessary for the greatest effect, or which mechanisms can be enhanced to improve effectiveness [9, 11]. Most exercise interventions are poorly specified, which makes replication often impossible [12, 13], but also limits the ability to decompose the exercise components. Further, exercise treatment targets are poorly specified in trials testing exercise for persistent LBP [10, 14]. The heterogeneity between exercise settings, types, prescribers, components, dosages, and durations [15], in addition to poor exercise intervention reporting means it is difficult to identify the active treatment components of exercise interventions.

### Justification for review

A realist review is an interpretative, theory-driven approach to synthesise evidence using multiple sources. The approach is increasingly used in the evaluation of complex interventions, such as therapeutic exercise, to enable a review of the broader evidence about the context of the intervention [16]. Realist reviews aim to understand why an intervention is effective, through which mechanisms, for whom and in what contexts, to impact outcomes of importance [17]. Realist reviews explore how underlying mechanisms (M) may be active in the context (C) of certain situations, environments, settings, or populations to create intended or unintended outcomes (O) [17]. For example, a person with LBP who has stopped all physical activity (C) may participate in, or complete, an exercise programme (O) because they are fearful of worsening pain or disability (M). Content is described through Context-Mechanism-Outcome (CMO) configurations. CMO configurations can be linked and allow for chains of possible theories to explain why a particular outcome occurs with a specific intervention. Interlinking CMO configurations can be further linked to form a ‘programme theory’ or model of how an intervention may work [16, 18]. Realist methods allow for a wider range of evidence sources to be included, in contrast to traditional systematic reviews (e.g. Cochrane reviews) which most commonly include RCT data only, precluding the ability to accommodate the complexity that may guide future improved prescription of exercise interventions. To date, there are no realist reviews of exercise interventions for the treatment of persistent LBP. Realist reviews have been conducted on exercise in other conditions such as frailty [19], physical activity in children [20], as well as older adults with cognitive impairment [21].

Although over 400 randomised controlled trials of exercise for persistent LBP have been published, there remains a lack of understanding of how therapeutic exercise prescription changes the outcomes of importance to people with persistent LBP [9]. It is important researchers and clinicians understand the overall programme theory of how therapeutic exercise prescription may create an effect on outcomes of importance, in order for potential mechanisms to be measured and targeted in research studies and in clinical practice. This will help to identify which components of exercise prescription are most important for future refinement and evaluation. The aim of the realist review is to build explanations and provide a deeper understanding of how and why therapeutic exercise prescription achieves their specific effects [22].

### Objectives of and focus of the review

The objectives of this review are (i) to identify the underlying programme theory of therapeutic exercise interventions prescribed for people with persistent LBP, and (ii) to explore how and why exercise improves outcomes of importance for those with persistent LBP. We have chosen to keep the review questions broad regarding outcomes of importance for patients, as this acknowledges the difference between patient-relevant outcomes (such as patient-reported outcomes like low back pain intensity, physical function and health-related quality of life, satisfaction with treatment and so on) as well as exercise engagement outcomes such as exercise adherence, participation in therapeutic exercise and so on.

### Research questions

The aim is to understand how therapeutic exercise creates change in outcomes of importance for those with persistent LBP. In particular, what components of exercise prescription, under what circumstances, improve outcomes, to what extent and why?

The research questions for the review are the following:

1. How does therapeutic exercise prescription create change in outcomes of adherence, engagement, and clinical outcomes for patients with persistent LBP?
2. What are the key behavioural mechanisms of exercise prescription?
3. Under what contexts is exercise prescription optimised?

## Methods

This review uses the five practical stages of review as described by Pawson [18]. This is not linear, and the reviewer is expected to move between stages to achieve theoretical saturation. These stages include (i) articulating key rough theory programme theories to be explored, (ii) searching for relevant evidence, (ii) appraising the quality of evidence, (iv) extracting the data, and (v) synthesising evidence [18]. The protocol will be reported according to the PRISMA-P guidance [23].

### Development of stakeholder groups

A stakeholder group was created using advertisements through the local patient and public involvement and engagement groups, the physiotherapy department, and approaching known experts in the field of chronic pain. People with experience in using exercise to treat persistent LBP were eligible to participate, as were healthcare practitioners involved in the management of people with persistent LBP. This led to the creation of a group including people with experience of using exercise to treat persistent LBP, exercise prescribers such as physiotherapists and personal trainers, and a psychologist. This led to the creation of a group including people with experience of using exercise to treat persistent LBP, exercise prescribers such as physiotherapists and personal trainers, and a psychologist. Consultation and discussion within this group were completed throughout the initial stages via two virtual meetings to agree on the rough programme theory. Notes from the meetings and audio recordings were used to refine the rough programme theories on a PowerPoint document to allow transparency of amendments.

A steering group informed the development of the research protocol and research funding application and met at regular intervals. The steering group comprised a Professor of Information Systems (AB), a Professor of Musculoskeletal Health and a physiotherapist (NEF), a Professor of Psychology Applied to Rehabilitation, a psychologist and physiotherapist, (SD), the lead Cochrane Review author of exercise and LBP and a chiropractor (JAH), a post-doctoral physiotherapist who had experience of performing realist reviews (VB), and a post-doctoral physiotherapist with clinical expertise as well as previous quantitative research experience in exercise prescription and persistent LBP (LW).

### Articulation of key theories

**Fig. 1:**
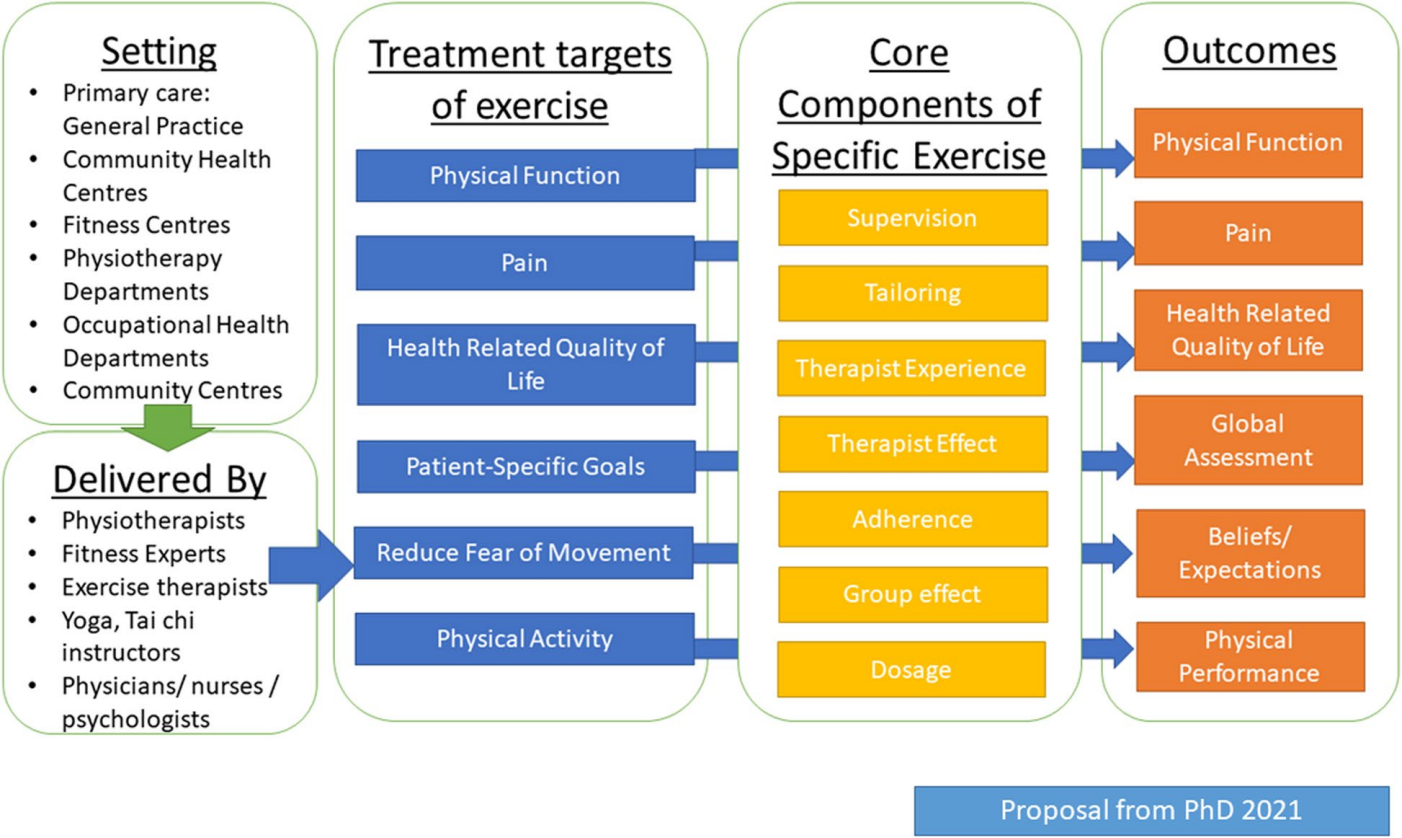
Initial programme theory taken from [10, 24]

**Fig. 2:**
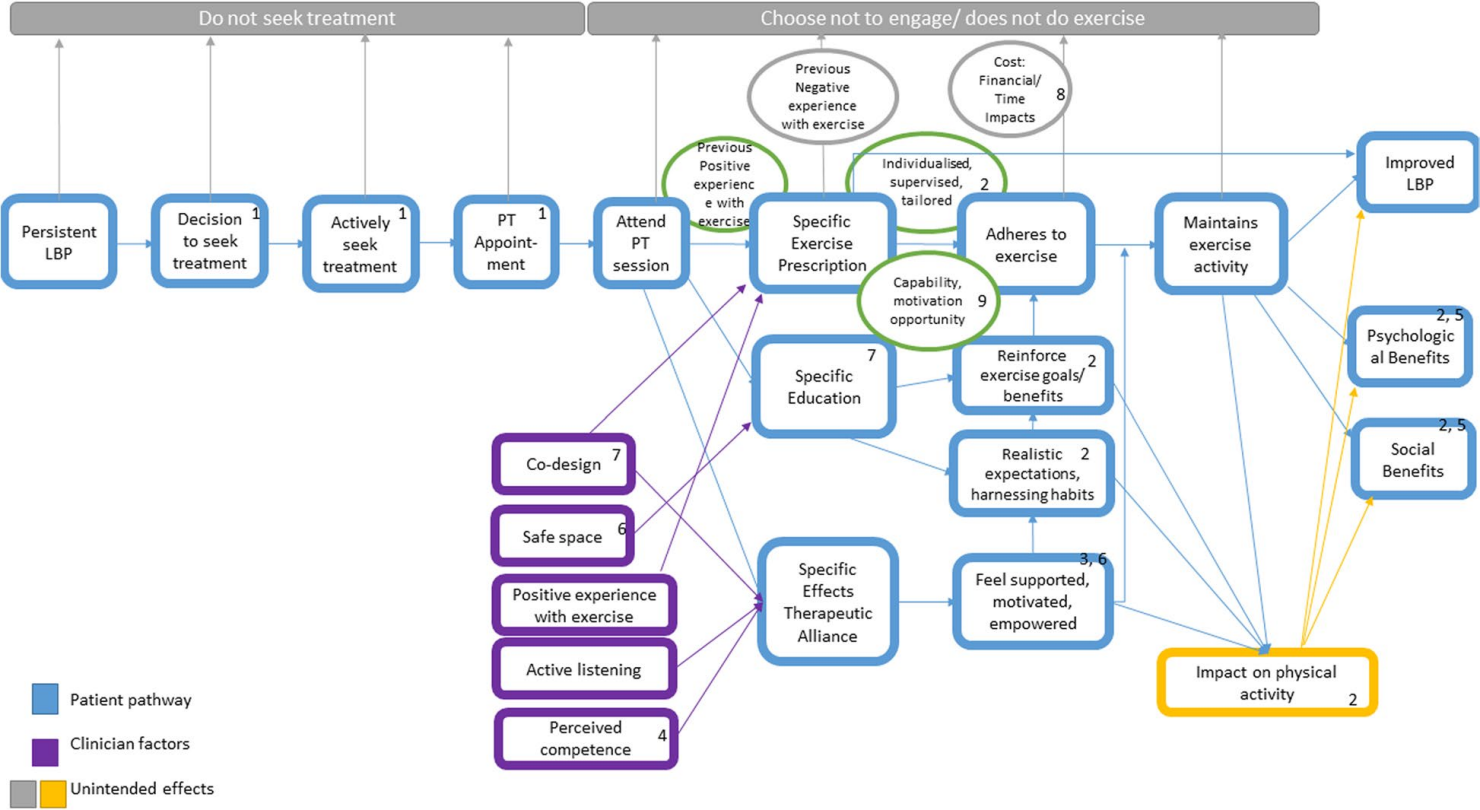
Iteration 2 of programme theory after initial scoping review and stakeholder meeting

**Fig. 3:**
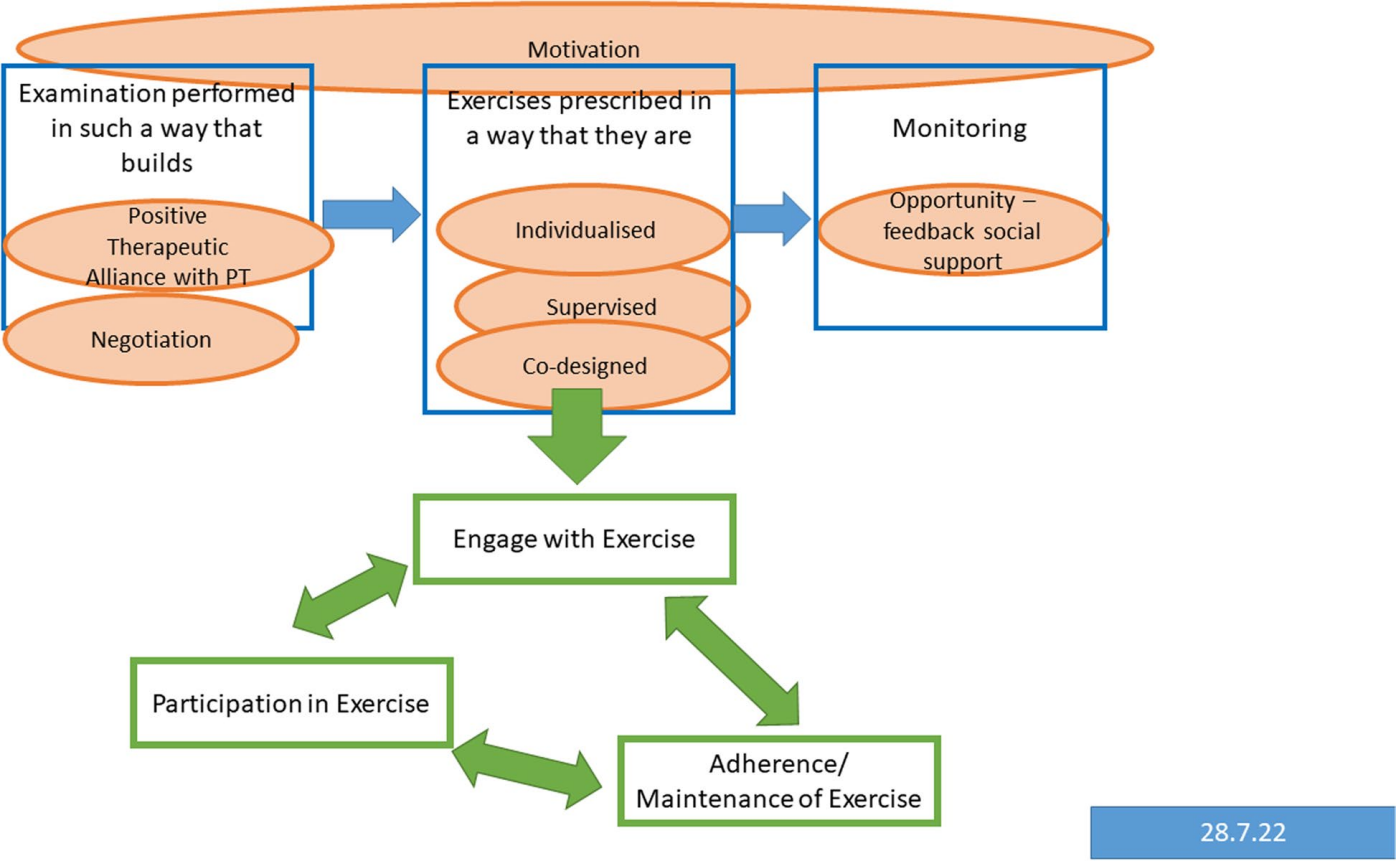
Initial draft programme theory

Exercise is a complex intervention, with many components that may influence the effect, adherence, and continued engagement, according to both individual patient and prescriber characteristics [15]. Initial programme theories were developed: firstly, during the completion of the lead researcher’s PhD (see Fig. 1) [24]. This initial logic model was updated further to encompass the patient journey from onset of persistent LBP to engagement or non-engagement with exercise, using initial scoping searches led by the lead researcher, discussion with realist experts during a taught course (Wong, Doddy) and structured scoping searches performed by an information specialist (Figs. 2 and 3). These were performed using broad search terms including the following: LBP, exercise, and mechanisms. This was an iterative process based on prior knowledge of the literature in exercise and LBP, and clinical experience. The initial Figs. 1 and 2 were presented to both the stakeholder and steering groups for discussion over 3 meetings and were refined to resemble Fig. 3, which were the agreed areas of thematic focus for the structured realist search. The CMO configurations were developed using Fig. 3 to provide a rough programme theory for further refinement and testing using the realist searches.

The above process identified 8 draft CMO configurations contributing to one rough programme theory to test against the literature summarised below:

When a patient with persistent LBP has a comprehensive, biopsychosocial assessment (context (C)), a positive therapeutic alliance will be created (C), and using negotiation (mechanism (M)) to decide on treatment planning, the patient is involved in co-designing (C) an individualised, supervised, exercise programme (C), and they are provided with monitoring through follow-ups and feedback (C) to provide sustained motivation (M) for engagement in exercise (outcome (O)), participation with the exercise prescription (O), adherence to exercise prescription (O) and improvements in patient-relevant outcomes (O).

#### The therapeutic consultation

(A) When a person with persistent LBP builds a positive therapeutic alliance (C), then they will be motivated (M) to engage with the prescribed exercise (O)
(B) When a patient with persistent LBP is assessed thoroughly by a (perceived) competent therapist (C), they are more likely to have more confidence in their therapist’s advice (M), which will facilitate their engagement with prescribed exercise (O)

#### Exercise prescription components

(III) When a patient with persistent LBP is prescribed an individualised exercise programme (C) that resonates with their goals (M), they are more likely to engage with the exercise (O)
(IV) When a patient with persistent LBP is prescribed exercises (C) that are co-designed, they will be more willing (M) to engage with the exercise (O)
(V) When a patient with persistent LBP is supervised while performing their exercise (C), they will have increased confidence in performing their exercises (M), which will increase adherence (O)
(VI) When a patient with persistent LBP has had a previous positive experience with exercise (C), then they may be more motivated (M) to engage with prescribed exercises (O)

#### Follow-up and monitoring effects

(G) When monitoring is provided in the form of follow-up appointments (C), this may allow the opportunity for feedback (M) and will positively encourage ongoing participation in the exercises (O)
(H) When peer support is provided in the form of group engagement (C), then this provides motivation (M) for continued participation in exercise programs (O)

### Database searches

This review will benefit from the guidance of an Information Specialist (AB) to ensure our search strategy is inclusive of all potentially relevant evidence. We will not limit the search to an arbitrary time period but will ensure our search is targeted and comprehensive to capture diverse mechanisms as identified over time. We will undertake an interactive and iterative search strategy. The realist review will use the guidance from Pawson [18] and Booth et al. [25]. Three databases will be searched (Ovid MEDLINE, Ovid PsycInfo, Ovid CINAHL) and an example search strategy is included in Supplement 1.

### Data selection

Data selection will be based on relevance, richness and rigour [26]. Selection of studies for inclusion will be based on relevance to the research aims and will provide information to support the development or testing of programme theory development. The studies retrieved will be considered for relevance using an assessment of the “fit” of the article to the research question, in particular, whether they focus on (1) therapeutic exercise, (2) persistent LBP (3), from the patient perspective. Material will be excluded if it is not published in English or does not focus on exercise for persistent LBP in adults. Reasons for exclusion will be documented and a second researcher will verify initial verdicts on eligibility. Potentially relevant full texts will be obtained. Available full texts will be independently screened by one member of the review team to confirm inclusion. Confirmation of full-text selection will be tested on a random sample of 10% by the stakeholder group, prior to proceeding with data extraction. To determine inter-rater agreement, a Kappa measure of agreement will be calculated at (1) the abstract review stage (two reviewers) and (2) the full article review stage (10% stakeholder review). Any discordant opinions or full-text studies will be reviewed a second time and further disagreements about study eligibility will be resolved through discussion with a third review author.

### Data extraction

Data will be extracted based on relevance to the aims of the review and the rough programme theory. Data will be sought that substantiates, refines, or refutes the programme theories and describes contextual characteristics. Data will be extracted to explain contexts (e.g. therapist or patient-specific features), mechanisms (e.g. motivation) and outcomes (e.g. adherence) and their influence on the CMO configurations recorded through annotations. Relevant studies will be highlighted, labelled, and recorded via NVivo software and Excel. A coding frame will be developed through discussion with the stakeholder group, based on the programme theory.

Independent reviewers will extract data through a standardised data extraction table, using a logic framework to guide extraction themes. Two reviewers will pilot test the data extraction table (LW and AB) with 10% of articles. Discrepancies will be discussed and resolved before proceeding with data extraction of the remaining studies.

Extracted data relevant to the review will include: study characteristics, participant characteristics; outcome measures used; programme theory configurations: (A) context (user type, setting of exercise delivery); (B) outcomes; and (C) mechanisms (barriers, facilitators, process of implementation); and, where relevant, intervention characteristics: (1) rationale; (2) materials; (3) provider; (4) type of exercise; (5) how delivered; (6) supervision component of delivery (7) motivational strategies used in delivery; (8) home exercise programmes; (9) adherence measurement; (10) setting of delivery; (11) when and how much; (12) tailoring; and (13) assessment of adherence and delivery as planned.

### Risk of bias (quality) assessment

Following realist review methodology and the RAMESES materials [27], the assessment of relevance and rigour of the studies will be used to determine the quality of the included texts [27]. Included studies will be scored on a five-point scale (where 1 = none whatsoever, 2 = poor, 3 = fair, 4 = good, 5 = exceptional) resulting in three scores: one for their relevance to the research questions, one for their rigour, one for their richness [25] (see Table 1 for more detail) [26]. Studies may be prioritised but will not be excluded based on these scores.

**Table 1:** Table summarising the rating scale criteria used for each of the quality assessments

Quality appraisal	Rating scale criteria
Richness	5 = Conceptually rich: studies with well-grounded and clearly described theories and concepts
	4 = Conceptually thick: studies with a rich description of a programme, but without explicit reference to the theory underpinning it
	3 or less = conceptually thin: studies with weak programme descriptions where discerning theory would have been problematic [26]
Relevance	5 = directly relevant if they focussed on low back pain, exercise prescription, therapeutic alliance, and the patient’s perspective
	4 = mostly relevant if they had one theme less than above
	3 = not directly relevant if the articles focused only on exercise or low back pain (not both) without additional information on patient perspectives or therapeutic alliance
Rigour	“Are the methods used to generate the relevant data are credible and trustworthy”
	5 = well-conducted RCT, well-conducted SR of RCTs
	4 = well-conducted quantitative studies, SR of other study types, well-conducted qualitative studies or mixed-method studies
	3 = RCT with concerns of bias, quantitative analysis with some concerns over methods
	2 = poor methodology (of any of the above) or not generalisable (case reports/case series)
	1 = non-research

*SR* systematic reviews, *RCT* randomised controlled trials

### Strategy for data synthesis

Data will be tabulated and synthesised to describe the included studies. We will conduct a thematic narrative approach to synthesise data using a data matrix. The data matrix will be formed according to context, mechanism, and outcome categories. Once data have been extracted and cleaned for consistency, they will be synthesised according to similarities and themes within these categories. Extracted data from the included studies across study designs will be combined and treated as textual (qualitative data). Synthesis of the data will occur in an iterative, complimentary process as the researcher (LW) is engaging with and extracting data. A process of charting, categorising and thematic synthesis of the extracted intervention components and qualitative data will be used to identify individual elements of the model. A key part will be detailing mechanisms of change within the patient pathway. One reviewer (LW) will familiarise themselves with the results of the included studies, and systematically and comprehensively assess the results of each study, highlighting important study characteristics and findings. The data coding and mapping will be checked, and discrepancies arbitrated by one to two additional reviewers as required. Preliminary mapping will be discussed with the wider research team and co-advisory panel. Revisions will be made based on feedback. Data analyses will be performed using Microsoft Word documents and Microsoft Excel spreadsheets.

### Theory testing

During theory testing, we will seek to validate the refined CMO configurations with further evidence. Secondary searches will be undertaken using excluded studies, reference list checks, and specific searches using Google Scholar and Pubmed. We will extract qualitative data that supports or refutes the CMO configurations, and quantitative data that may summarise the effect of a CMO configuration. For quantitative data, we will extract the overall certainty of results where synthesis had incorporated Grading of Recommendations, Assessment, Development and Evaluations (GRADE) framework recommendations [28]. We will extract the meta-analysed between-group mean differences or odds ratios (with 95% confidence intervals (CIs) or credible intervals (CrIs) as reported) or single study data as available.

### Data analysis

Codes will be allocated to elements of the rough programme theory and iteratively adapted according to new material. The initial programme theories will be refined using the literature, and then tested with quantitative and qualitative evidence to support the statements. Where exercise interventions are described, these will be analysed with respect to the treatment targets identified, dosage, duration, and type of delivery of exercise intervention, as well as the content included alongside the exercise intervention.

The final programme theory will use the graphical presentation to illustrate the chain of reasoning underpinning how components of exercise produce mechanisms that lead to immediate (or short-term) outcomes and then to longer-term outcomes and impacts [29] This lays out the proposed components that underpin the pathway (in this case, how exercise creates a change in LBP outcomes of importance). The stakeholder group will be involved in key stages of interpreting and analysing the results.

### Strength of findings

Using the GRADE-CERQual system, [30] we will assign certainty to each of the CMO configurations based on the rigour, relevance, coherence, and adequacy scores of the included studies providing evidence for each statement where possible.

### Output

The results of the realist review will be visually mapped into a programme theory or logic model to describe the proposed CMO configurations. The review will be reported according to the Realist and Meta-narrative Evidence Syntheses: Evolving Standards (RAMESES) publication standards [27] and the PRISMA-P statement (see Supplement). A summary of the review findings will be included in a report to the funding organisation (Orthopaedic Research UK). The results of this review will be published in a peer-reviewed journal and disseminated at international and national conferences. The stakeholder group will be actively involved in aiding dissemination via non-academic routes, with lay summaries for patient and consumer groups (e.g. BackCare and the UK Spine Society Board), as well as dissemination via local National Health Services (NHS) Trusts, and physiotherapy networks. This review will be the first, to our knowledge, use of realist review methods in exercise for LBP; therefore, the process as well as the review findings will be included in any presentation or dissemination process.

### Ethical issues

Ethical approval is not required to conduct this review. Consent and agreement of discussion documentation were sought from the stakeholder group regarding the development of the rough programme theories. Prior to funding, the project protocol was reviewed by Clinical academics and lay members of the Orthopaedic Research UK charity. The review has been registered with PROSPERO (CRD42017072023).

## Discussion

In the field of persistent LBP research, it remains unclear as to what components of therapeutic exercise prescription lead to optimal outcomes of engagement, participation, adherence and overall improvement. Exercise for persistent LBP is proposed to work through five different mechanisms: psychosocial, neuromuscular, neurophysiological, cardiovascular and tissue healing [11]. Most RCTs appear to favour neuromuscular treatment targets of strength and flexibility [10, 14]. However, no correlation exists between these favoured neuromuscular pathways and changes in pain and physical function [31, 32]. Mediation analyses in RCTs of persistent pain suggest that irrespective of treatment or condition, similar mechanisms of effect exist, and appear to be primarily psychological [33]. Increasingly, the non-specific effects of the therapeutic alliance [34–36], and the placebo effects [37] of treatment consultations are recognised as important in therapeutic exercise prescription. The psychological processes and mechanisms involved in these therapeutic components (beliefs, confidence, motivation) may account for a degree of the benefit recorded [37, 38]. In our consensus workshops with multiple stakeholders including international experts, during which treatment targets were considered, the core outcomes of physical function, quality of life and pain were highly ranked, followed by psychosocial targets of patient-specific goals, fear-avoidance, self-efficacy and attitudes, thoughts and beliefs [24]. In exercise for persistent LBP, the optimal pathway through which therapeutic exercise prescription creates change is unclear at present. Without a clear understanding of which treatment targets drive change in outcomes of importance, it is difficult to optimise the therapeutic effect of exercise in clinical and research settings. Thus, our team hypothesised that these psychosocial targets may account for a considerable proportion of how exercise changes outcomes. This review aims to unpack the black box of therapeutic exercise prescriptions for persistent LBP and describe how some of these behavioural mechanisms may influence outcomes.

The strength of this review is the multi-disciplinary team of researchers, including an information specialist (AB) to lead the realist review search strategy. Realist review methods encourage transparency about the researcher’s influence on certain aspects, such as the initial rough programme theory development and interpretation of the literature. In this realist review, the lead researcher’s knowledge as a clinician and researcher in the field of persistent LBP assists the theory development by adding detailed recognition of potentially ‘hidden’ mechanisms at play, and a deeper understanding of the CMO configurations. This contrasts with traditional systematic review methods, where the researcher is considered a potential source of bias. The stakeholder group, second reviewer and steering group provide consistency and transparency in decision-making and ensure the clinical relevance and focus of the review. This review is limited by time and resources as it is a funded project of 24 months. To ensure timely delivery, this may limit the depth and detail that the review can achieve. Finally, the published materials in the LBP field may not include the level of theoretical reasoning required to adequately test and define the programme theories. Research into LBP has largely favoured quantitative research methods and syntheses that follow a rigid reporting structure. Many interventions are poorly described despite reporting checklists such as the CERT and TIDIER [12]. Thus, it is expected that the search strategy and subject field may be adapted as the review progresses.

## Summary

Exercise treatments for persistent LBP are associated with lower healthcare system costs and improvements in quality of life when compared to usual care [39]; however we are still unclear about how therapeutic exercise prescription creates change in outcomes of importance [11, 24]. Realist reviews may provide additional information to traditional systematic review methods. This can aid our understanding of the contextual features that may influence exercise prescription, such as for whom they are most effective, in what setting, how they are implemented and why. This realist synthesis will enhance our understanding of therapeutic exercise prescription to improve adherence and engagement and ultimately will provide clinically relevant recommendations regarding exercise prescription for persistent LBP.

### Supplementary Information

**Additional file 1: Supplement 1.** PRISMA-P (Preferred Reporting Items for Systematic Review and Meta-Analysis Protocols) 2015 checklist: recommended items to address in a systematic review protocol* Section and topic.

## Data Availability

The final review results will be published and available.
